# Supplementary material for: Serial founder effects slow range expansion in an invasive social insect
Source: Nat Commun. 2024 Apr 29;15:3608. doi: 10.1038/s41467-024-47894-1 (PMC11058855; doi:10.1038/s41467-024-47894-1)
Supplement: Supplementary file 1 — Supplementary Information [file 41467_2024_47894_MOESM1_ESM.pdf]

Supplementary Information

Serial founder effects slow range expansion in an invasive social insect

Supplementary Methods

1.1 Model Overview

We developed an agent-based model to examine how allele frequencies at the sex locus (and thus genetic load) vary over time and space in a colonizing population of honey bees. This model used a single, continuous spatial dimension along which agents (colonies) reproduced and dispersed in discrete generations, expanding in two directions from the point of incursion. Each colony was assigned a fitness score according to the proportion of diploid males (DMP) it produced (i.e. according to the sex alleles present in the queen and her mates). This fitness score was used to determine, per colony, both the reproductive output and the likelihood of survival until the next generation.

The model had three phases that comprised a discrete generation: reproduction, dispersal and persistence. During the reproductive phase, colonies produced virgin queens and drones, according to their fitness score. These then mated with neighbouring virgin queens and drones to produce new colonies. During the dispersal phase, colonies stochastically moved to a new position on the single spatial dimension. Each colony was capable of moving in both directions along the spatial axis, however we considered scenarios where movement was somewhat biased towards regions of lower density to be most likely given that suitable nesting sites are likely to be a limited resource for cavity-nesting honey bees [1]. Finally, during the persistence phase some colonies persisted while others perished (i.e., were removed from the simulation) based on their fitness score, colony density and age.

Our first goal was to check that our simulated populations showed increased genetic load at range edges, consistent with our empirical dataset of Australia's invasive *Apis cerana*. We therefore set most parameter values to match best estimates for this population and/or honey bees generally: *csd* allele number and starting frequency (Gloag et al 2017), mean number of queen mates [2], proportion of colonies that go queenless each generation [3], maximum flight distance of drones [4], maximum numbers of drones produced in each colony [5], distance scout bees fly to find suitable nesting sites [6] and maximum age of queens [7, 8]. For four parameters, however, estimates from empirical data are not available and we considered that a range of values were plausible: the impact of diploid male production on colony

reproductive output and survival (i.e. the fitness function for DMP; Linear or Sigmoid; **Figure S1**), average reproductive output (i.e. the average number of swarms produced by a colony with no DMP; S_A = 2, 3 or 4), strength of dispersal (i.e. the tendency for new colonies to locate a nest site in areas with fewer other colonies vs. more other colonies; β = 0, 5 or 10), and maximum population density (i.e. the maximum number of colonies that can occur per unit space of our linear simulated population; K = 30, 100 or 300 colonies/unit space). Each of these parameters are described in detail below. In these cases, we performed a sensitivity analysis to confirm that the simulation results were consistent across all combinations of parameter values. In total, for each set of these parameters (54 combinations) we ran 96 simulations, each for 20 generations. To account for the populations expanding outwards in two directions, analysis of the simulations used the absolute value of the distance a colony was found from the point of incursion (see also Figure 2D of the main text).

Our second goal was to use this model to compare the rate of population expansion in populations where range-edge founder effects lead to genetic load at the sex locus vs. those where no such genetic load occurs; that is, to assess the consequences of reduced fitness at range edges on the rate of population expansion. To do this, we repeated a subset of the simulations but made the simulated locus fitness-neutral, (i.e. no cost of homozygosity at the locus), by setting all fitness scores to be equal to 1. This subset of simulations considered all values of dispersal strength (β = 0, 5, 10), as this parameter was found to have the largest impact on the results of the simulation, but fixed values for the DMP fitness function, average reproductive output and maximum population density (Sigmoid fitness function, S_A = 3 and K = 100; as these parameters had little impact on model outputs). By comparison with our previous simulations, this “fitness neutral” scenario allowed us to determine the change in range expansion rate that could be attributed to genetic load at the sex locus alone. Our model was written and run in R version 4.0.4, and the code can be found on github (https://github.com/Thomas-Hagan/BeeSimulation.git).

1.2 Reproductive Phase

Population Initialisation (generation 1)

For each colony, parameter values were stored in the dataframe $\mathbf{Q}$, like so;

$$\mathbf{Q} = \begin{bmatrix} p_1 & \tau_1 & f_1 & q_1^a & q_1^b & A_1^1 & A_1^2 & \cdots & A_1^k \\ p_2 & \tau_2 & f_2 & q_2^a & q_2^b & A_2^1 & A_2^2 & \cdots & A_2^k \\ \vdots & \vdots & \vdots & \vdots & \vdots & \vdots & \vdots & \ddots & \vdots \\ p_i & \tau_i & f_i & q_i^a & q_i^b & A_i^1 & A_i^2 & \cdots & A_i^k \end{bmatrix}$$

where p_i was the position of the colony, τ_i was the age of the colony's queen, f_i was the fitness score of the colony, q_i^a was the allele of the queen inherited through her mother, q_i^b was the allele inherited through her father, and A_i^k was the number of mates the queen had containing the *k*th allele in the population. In all cases, i denotes the *i*th queen in that generation.

We assumed a starting population (generation 1) of N = 11 colonies placed at a regular interval (0.2) between the initial start positions (-1 to 1). This population started with seven sex alleles at unequal frequencies (five equally-common alleles and two rarer alleles; an approximation of the Australian *Apis cerana* population in the first years post-invasion; Gloag et al 2017), and each queen was assigned two of these seven alleles according to these frequencies. To achieve this, queens were randomly allocated alleles such that A^6 and A^7 each made up approximately 2.6% of the total queen alleles in the population, with other alleles (A^{1-5}) having approximately equal frequency. Each queen in generation 1 then mated with 29 drones (the mean mate number for *A. cerana* queens; Ding et al 2017), where mates' sex alleles are assigned randomly such that A^6 and A^7 each made up approximately 10% of the total drone alleles in the population and A^1- A^5 had approximately equal frequencies.

Here we assumed that the fitness of a colony was negatively proportional to its percentage of diploid male production, unless otherwise specified. Therefore, we calculated the fitness score (f_i) of each colony as;

$$f_i = 1 - \frac{1}{2}\left(\frac{A_i^x + A_i^y}{\sum A_i}\right) \tag{S1}$$

where $x = q_i^a$ and $y = q_i^b$. This produced a value bound between 0.5-1 and corresponded to the proportion of healthy worker brood within a colony (i.e. *non*-diploid male offspring).

Finally, all values of y_i and ρ_i are initialised at 0.

Subsequent Generations

Subsequent reproductive phases consisted of two steps: (i) creating new virgin queens and (ii) mating those virgin queens. Virgin queen production began by determining how many new swarms each colony produced (where swarms are daughter colonies that bud from the parent colony, each headed by a new queen). The number of swarms produced was calculated according to a Poisson distribution like so:

$$f(x) = \frac{e^{-\lambda}\lambda^{x}}{x!} \tag{S2}$$

where x was the number of swarms produced, $f(x)$ was the probability of x swarms being produced, and λ was both the mean and variance of the distribution. Under simulations with linear fitness functions, λ was determined like so:

$$\lambda = f_i S_A \tag{S3}$$

where f_i was the fitness score and S_A was the average number of swarms produced by a colony that was not influenced by the production of diploid males (in these simulations, S_A = 2, 3 or 4). For simulations assuming a Sigmoid fitness function, an additional step was used to determine the number of swarms produced per colony. In these Sigmoid simulations, the derived fitness score F_i was determined like so:

$$F_i = 0.5 + \frac{(1 - 0.5)}{(1 + e^{-25(f_i - 0.75)})} \tag{S4}$$

Equation (S4) produces a sigmoid curve centred on DMP = 0.25, such that colonies with low proportions of diploid male production had F_i of almost 1, colonies with high proportions of diploid male production had F_i close to 0.5 (**Supplementary Figure 3**). Using this, we determined λ (and thus the number of swarms produced per colony, according to equation (S2)) in the case of the Sigmoid fitness function as:

$$\lambda = F_i S_A \tag{S5}$$

In both equations (S3) and (S5) (i.e. for both fitness functions modelled: Linear or Sigmoid) colonies with lower fitness scores produced fewer swarms on average, but there was still variation in reproductive output between colonies with the same fitness score.

One complication of honey bee biology is that while queens produce all diploid brood and most haploid (male) brood, workers can also lay haploid (male) eggs under some conditions. In Australia's *A. cerana* population, queenless colonies producing males are common, presumably because young queens leave with secondary swarms but then die during their mating flight (Gloag et al 2019). This high rate of worker reproduction might increase the

persistence of sex alleles in a local population (Gloag et al 2019). Therefore, after each colony in our simulation had produced a number of swarms, we determined whether each swarm became queenless with a probability of 0.3 (the queenlessness rate previously estimated in this population; Gloag et al. 2019). Any swarms that were queenless were removed from dataframe Q and added to a queenless dataframe as a copy of its parent colony, so that we could later produce drones only from these colonies using worker genotypes (i.e. workers in those colonies contribute to the male gene pool). Otherwise, each virgin queen inherited one allele from its mother and one allele from its father (one of its mother's mates). Any "queens" that had inherited two copies of the same allele were removed from the simulation (as these had the lethal diploid male homozygous condition).

To then mate the new virgin queen, we simulated a male mating pool comprising drones from neighbouring colonies. The list of neighbouring colonies included established colonies and queenless swarms (henceforth collectively colony *j*) over the following range (R_M):

$$R_M = \{x \in \mathbb{R} : (p_i - d_f) \leq x \leq (p_i + d_f)\} \tag{S6}$$

where d_f was the maximum distance drones can fly. As previous studies have indicated *A. mellifera* drones fly as far as 3.75 km from their natal colony to mate [4], we chose a conservative maximum flight distance of 5 km (d_f = 5); that is, we allowed for drone flight at the upper limit (whereas lower values would presumably exaggerate even further the founder effects at range edges). The number of drones (n_d) attracted to a virgin queen from colony *j* was proportional to the distance between the virgin queen and colony *j*, as well as the fitness score of colony *j*. We approximated this by finding the integral of a small section of a normal distribution, which we calculated like so:

$$n_d = D_c f_j \int_{p_i-\delta}^{p_i+\delta} \frac{1}{\sigma\sqrt{2\pi}} e^{-\frac{(x-p_j)^2}{2\sigma^2}} dx \tag{S7}$$

where D_c was the total number of drones that any surrounding colony with a maximum fitness score produces (f_i = 1, D_c = 1000; [5]), f_j was the fitness score of colony *j*, p_i was the position of the virgin queen, δ was the small area over which the normal distribution was integrated (δ = 0.5) and is analogous to the likelihood of drones arriving in the DCA occupied by the virgin queen, σ was the standard deviation of the normal distribution and stands in for the standard deviation of drone flight (σ = 2) and p_j was the position of the *j*th donating colony. After n_d was determined, each drone was assigned a sex allele according to its parent

colony *j*. If the colony was queenright, the sex allele was randomly chosen from the two queen alleles of colony *j*. If the colony was queenless then we simulated worker reproduction by assigning each sex allele a 50% chance of being a queen allele, or a 50% chance of being one of the alleles of the queen's mates (i.e. a paternal allele, proportional to the allele distribution of the queens mates). Where colony *j* had a large number of drones (>50), only the alleles of 50 drones were chosen randomly; others were assigned in equal proportion to the allelic makeup of the parent colony. We called the list of sex alleles produced from this process the "mate pool".

Despite large numbers of drones being present in a DCA, a virgin queen will only mate with a subset of the drones present. Therefore, we stochastically determined the number of drones a queen mates with using a probability function determined by a normal distribution with the following equation:

$$f(x) = \frac{1}{\sigma\sqrt{2\pi}} e^{-\frac{(x-\mu)^2}{2\sigma^2}} \tag{S8}$$

Where x was the number of mates, and where the mean (μ = 29) and standard deviation (σ = 8) were chosen based on true mate number observed in natural populations of *A. cerana* (Ding et al 2017). Where relevant, x was rounded to a whole number. If x was equal to or less than 0 it was set to 1, likewise if the mate pool was smaller than the value of x then x was instead set to the size of the mate pool. The mate pool was then sampled without replacement a number of times equal to x, and each allele was sorted into its relevant column in dataframe Q. Similar to the mating of drones and queens in nature, the number of drones that actually mated with a queen was exceedingly small. Therefore, it is highly unlikely that a simulated colony was ever exhausted of drones.

The age (in generations) of all queens was then increased by 1. All colonies of age 5 and all queenless swarms were then removed, simulating the death of old queens (honey bee queen lifespan estimates range from 2-3 years [8] to 8 years [7]) and queenless swarms respectively. The fitness score (f_i) of each new queen was calculated as per equation (S1) and the new colonies were appended onto data frame Q.

1.3 Dispersal Phase

The dispersal phase consisted of two sub-phases: (i) calculating the direction and strength of swarm movement and then (ii) explicitly generating the movement distance. This occurred for both established colonies (reflecting the movement of mature queens in the primary reproductive swarm of each swarming season) and newly created swarms (reflecting the secondary swarms headed by daughter queens). Throughout this section we refer to colonies undergoing movement as swarms.

The direction and distance of movement was calculated such that swarms stochastically dispersed away from the parent colony, but in some cases with a tendency to move (if available) towards regions of lower colony density. We simulated dispersion in this way as *A. cerana* is a cavity nesting species that will search for nearby free nesting sites during the swarming phase. This was dictated by two parameters, dispersal strength (β = 0, 5 or 10) and a movement pressure (γ_i), derived from the position and number of surrounding colonies. If β = 0, a swarm's movement was not impacted by γ_i, and swarms moved in a completely stochastic fashion with no tendency towards either direction.

To calculate γ_i, we considered neighbour colonies present on each side of the swarming colony *i* (r_s = 4, i.e. within 4km in either direction, a conservative estimation of maximum distance scout drones fly for nests during swarming [6]): positive (to the right) and negative (to the left). The position of positive and negative neighbour colonies were then assigned into two respective vectors (D_P and D_N). γ_i was then calculated as;

$$\gamma_i = \frac{(p_i - \overline{D_P})e^{\left(\frac{-|D_P|}{60r_s}\right)} + (p_i - \overline{D_N})e^{\left(\frac{-|D_N|}{60r_s}\right)}}{2r_s} \tag{S9}$$

where $\overline{D_P}$ was the mean of D_P, $|D_P|$ was the length of D_P and $\overline{D_N}$ and $|D_N|$ were likewise the mean and length of D_N. In cases where a vector was equal to, or had length, 0 the equation became;

$$\gamma_i = \frac{(p_i - \overline{D})e^{\left(\frac{-|D|}{60r_s}\right)}}{r_s} \tag{S10}$$

where D was the remaining vector. In cases where both vectors were equal to, or had length, 0 then the equation became;

$$\gamma_i = 0 \tag{S11}$$

Equations (S9), (S10) and (S11) result in γ_i ranging from -1 to 1. This function only took into account local neighbours, and therefore values of γ_i tended to be low. Such low values resulted in only minor deviations in the normal distribution's mean. Central colonies tended to have had values of γ_i close to 0 (that is, population density was equally high in both directions), as did swarms on the extreme range edge (where population density was equally low in both directions).

γ_i was then fed to a movement function that determined the explicit movement distance. This function generated a value $f(x)$ based on the probability function of a normal distribution:

$$f(x) = \frac{1}{\sigma\sqrt{2\pi}} e^{-\frac{(x-\beta\gamma_i)^2}{2\sigma^2}} \tag{S12}$$

where γ_i was the centre of the distribution, and β was the propensity to disperse (β = 0, 5 or 10), σ was the standard deviation of each movement (σ = 4), x was the distance the swarm moves and $f(x)$ was the probability of moving x distance. As movement was stochastic, all swarms, including those near range edges, potentially moved towards either the range centre or the range edge, but when β > 0 they were somewhat more likely to move towards range edges (i.e. lower density regions). The parameter value for β and σ were chosen as they result in a movement that approximates what we see in our natural population: a range expansion of approximately 7 km a year (for the case of β = 10). While β = 10 matches the real population of invasive *A. cerana* in Australia, we elected to model β = 5 and β = 0 to investigate whether this type of outward-biased dispersal impacts the simulation significantly. In particular, β = 0 models the population with no directional tendency in dispersal at all.

To prevent any swarm from moving an unreasonably large distance, each value of x was capped at a maximum absolute value of 10 (the estimated maximum dispersal distance for honey bee swarms; (Villa 2004). Lastly, x was added to p_i to give the colony's new position.

1.4 Persistence Phase

In the final phase of the simulation, some colonies die. As all aspects of colony function depend on the workerforce, we considered that the likelihood that a colony survived to the next reproductive phase depended on the proportion of diploid brood that were workers rather than inviable diploid males (i.e. the colony fitness score, f_i). That is, colonies with lower fitness scores were less likely to survive. As for the reproductive phase (1.1), we considered two possible fitness function for DMP (Linear or Sigmoid). In addition to f_i, the probability of survival in all cases also relied on the maximum colony density value (K) and the actual density of surrounding colonies. We included K in our simulations to prevent the number of colonies per unit space increasing *ad infinitum*. To confirm that our results were generalizable across different values of K, we ran our simulations at three different levels (K = 30, 100 or 300 colonies/unit space). K influenced the survival of a colony compared to other local colonies over the following range (R_S):

$$R_S = \left\{x \in \mathbb{R} : \left(p_i - \frac{1}{2}\right) \leq x \leq \left(p_i + \frac{1}{2}\right)\right\} \quad \text{(S13)}$$

Under the Linear fitness function, the f_i of colony *i* was compared to the average f of neighbouring colonies over the range R_S. Each colony *i* had a probability of survival equal to:

$$p = \frac{f_i K}{\omega_i |R|} \quad \text{(S14)}$$

Where f_i was the fitness score of the ith colony, K was the maximum population density and $|R|$ was the number of colonies in range R. ω_i was a normalisation factor, equal to the average fitness score (f) of all colonies surrounding the ith colony. The outcome of a linear fitness function was that when colony densities were well below K, all colonies were likely to survive (regardless of f) because p was almost always larger than 1. When colony densities approached or exceeded K, higher f colonies were more likely to survive than lower f ones. Instead, under the Sigmoid fitness function, colony i had a probability of survival similar to equation (S14), but where f_i was replaced by F_i (derived fitness score, equation (S4)):

$$p = \frac{F_i K}{\omega_{Fi} |R|} \quad \text{(S15)}$$

ω_{Fi} was the same normalisation factor used in equation (S14) but was calculated using the derived fitness score (F) of surrounding colonies instead of the base fitness score (f). This resulted in colonies biased towards low (0.5) or high (1) F_i, simulating the higher survival rates of colonies with small proportions of diploid male brood.

In all cases, colonies that were removed from the simulation were done so all at once at the end of their persistence phase. Once colonies had been through this phase, the generation was increased by 1 and the simulation returned to the reproductive phase.

2.1 Sensitivity analysis of model parameters: fitness function and maximum population density

Our simulations revealed that colonies at range edges had lower average fitness than those in range centre. This trend was consistent across parameter values: fitness functions (Linear or Sigmoid), average reproductive output (S_A = 2, 3 or 4), dispersal strength (β = 0, 5 or 10) and maximum population density (K = 30, 100 or 300). **Supplementary Figures 4-5** show illustrative combinations of these parameters.

We found that K did not impact the diploid male production of colonies in our simulations, with diploid male production at range centres and at range edges being relatively similar across all parameter permutations (**Supplementary Figure 4**). We also found that K did not have an impact on the distance of the range edge reached by generation 20 across parameter permutations. We elected to use K = 100 for our final comparison between simulations of a homozygous lethal and fitness neutral locus.

We found that the fitness function we simulated (linear or sigmoid; **Supplementary Figure 3**) did not impact the amount of diploid male production of colonies in our simulations, with diploid male production at range centres and at range edges similar across both functions (**Supplementary Figure 4**). The fitness function used did somewhat impact the average distance of the range edge reached by generation 20 across parameter permutations; the Sigmoid fitness function reached a range edge slightly further than the linear fitness function (approx. 2 units further). We elected to use the Sigmoid fitness function for our final comparison between simulations with a homozygous lethal and fitness neutral locus.

We found that S_A did not impact the diploid male production of colonies in our simulations, with diploid male production at range centres and at range edges being similar across parameter permutations (**Supplementary Figure 5**). However, we did find that S_A had an impact on the distance of the range edge reached by generation 20 across parameter permutations, with higher reproductive outputs resulting in larger distances (approx. ~12 units difference between each value of S_A). This result is not surprising, as the more colonies produced the higher the likelihood of range edge colonies producing swarms that pushed

range edges further with each generation. We elected to use a S_A = 3 for our final comparison between simulations of a homozygous lethal and fitness neutral locus.

We found that β had the largest overall impact among the parameters that we varied in our simulations (**Supplementary Figure 5**). While β had no impact on the diploid male production of colonies in the range centre, it effected the diploid male production of colonies at the range edge, with smaller values of β resulting in smaller rates of diploid male production at range edges (**Supplementary Figure 5**). Notably, despite impacting diploid male production at range edges, even when β = 0 we still found an increase in diploid male production at the range edge. Furthermore, β impacted the distance of the range edge reached by generation 20, with larger values of β resulting in larger distances (approx. ~15 units difference between each value of β). As this parameter impacted both diploid male production, and the distance the range edge reached by generation 20, we considered all three values of β = 0, 5 and 10 for our final comparison between simulations of a homozygous lethal and fitness neutral locus and found that our conclusions held for all values.

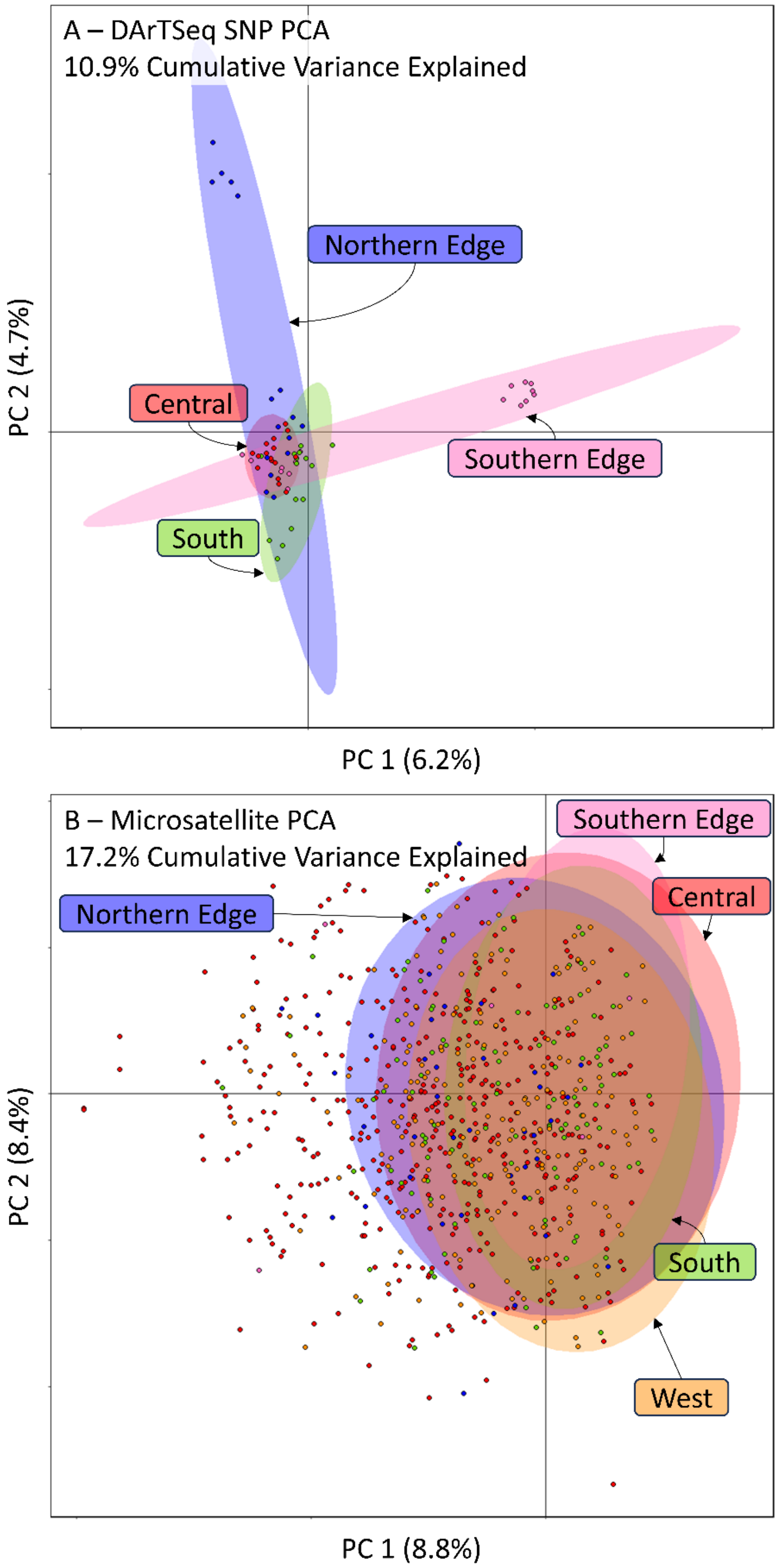

Supplementary Figure 1: **A** PCA of 2829 SNPs from 63 drones within Australia's population of invasive *A. cerana*, showing differentiation of northern edge and southern edge regions due to some individuals in each region falling outside the main cluster. **B** PCA of eight microsatellite (single sequence repeat, SSR) loci from 4639 drones (males) within Australia's population of invasive *A. cerana*, showing that individuals do not differentially cluster by region at these markers. Populations are coloured as followed; Northern Edge = Blue, Central = Red, West = Orange, South = Green, Southern Edge = Pink.

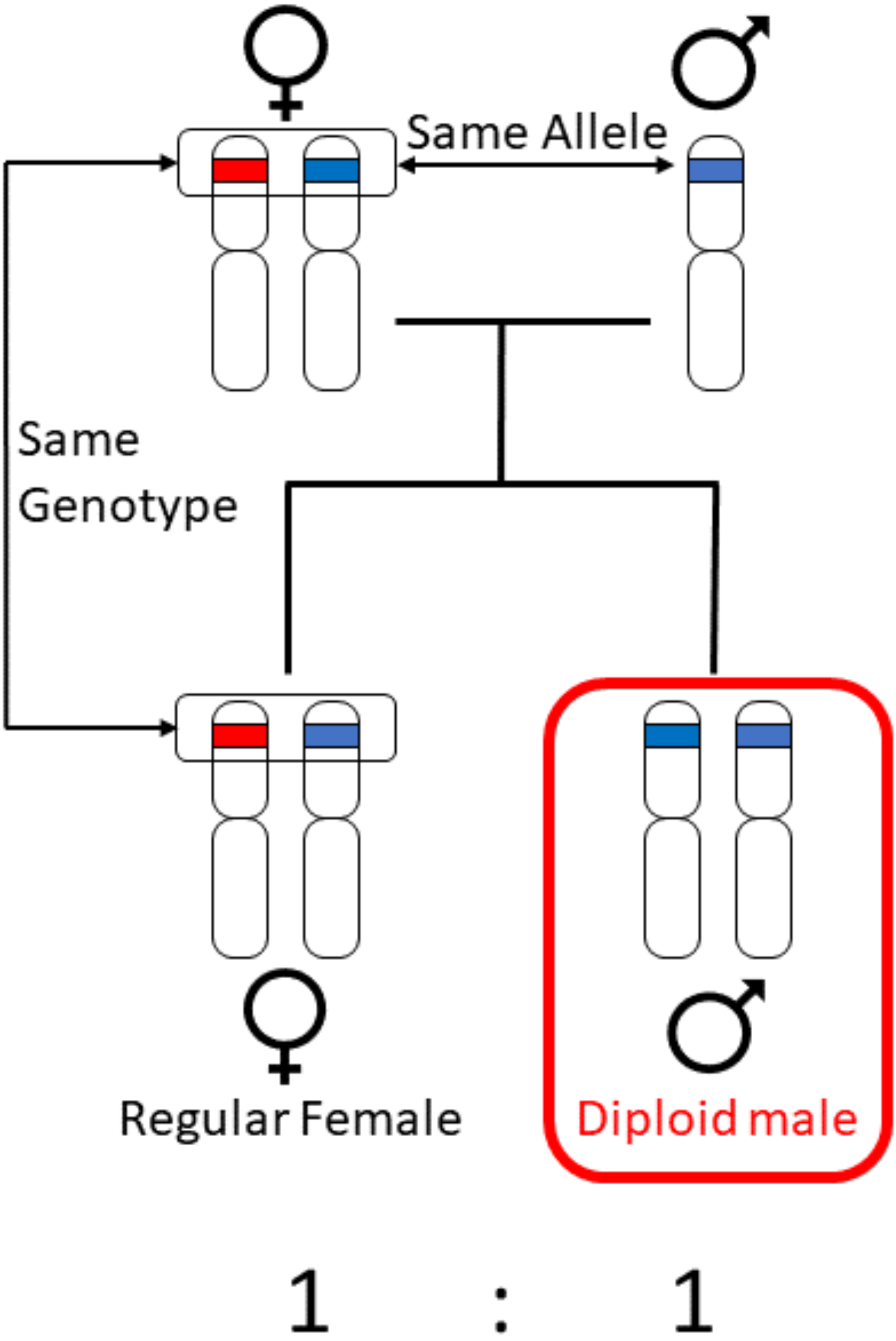

Supplementary Figure 2: A demonstration of the calculation of diploid male incidence per colony. Diploid male embryos are removed from the colony prior to adulthood but can be readily inferred from the *csd* genotype (represented here with coloured bands) of surviving female brood. Every worker that shares the same two *csd* alleles as the queen must equate to a (now deceased) diploid male in a ratio of 1:1.

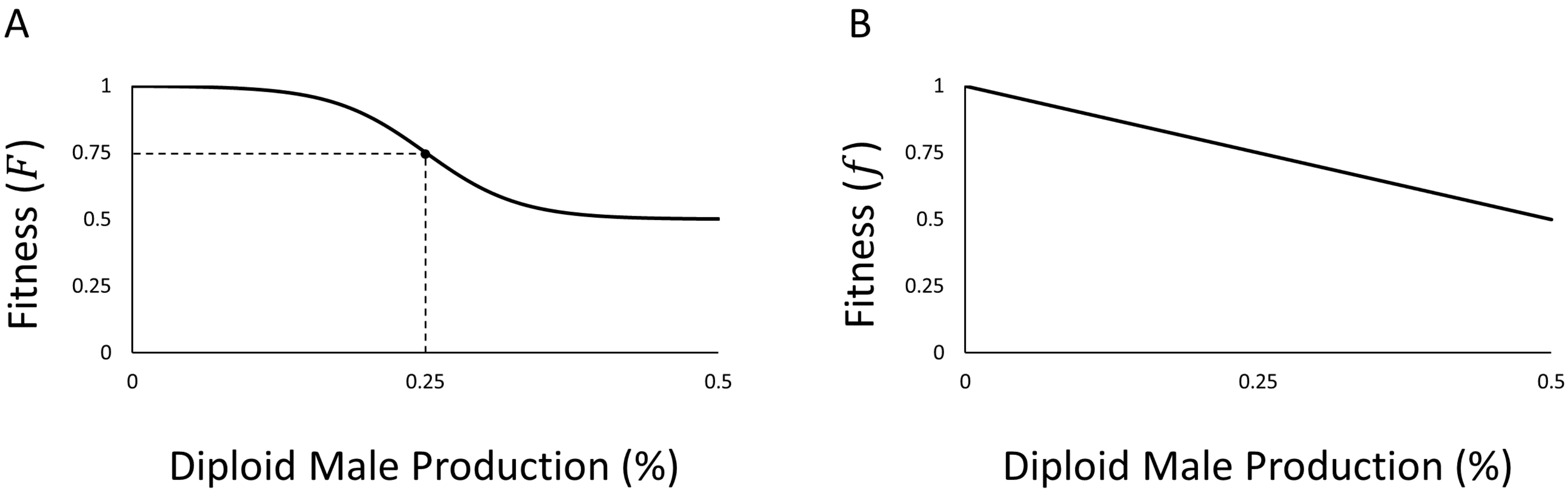

Supplementary Figure 3: A visual representation of the relationship between fitness (F or f) and the diploid male production of a colony used in our simulations where we assume **A** Sigmoid fitness function and **B** Linear fitness function.

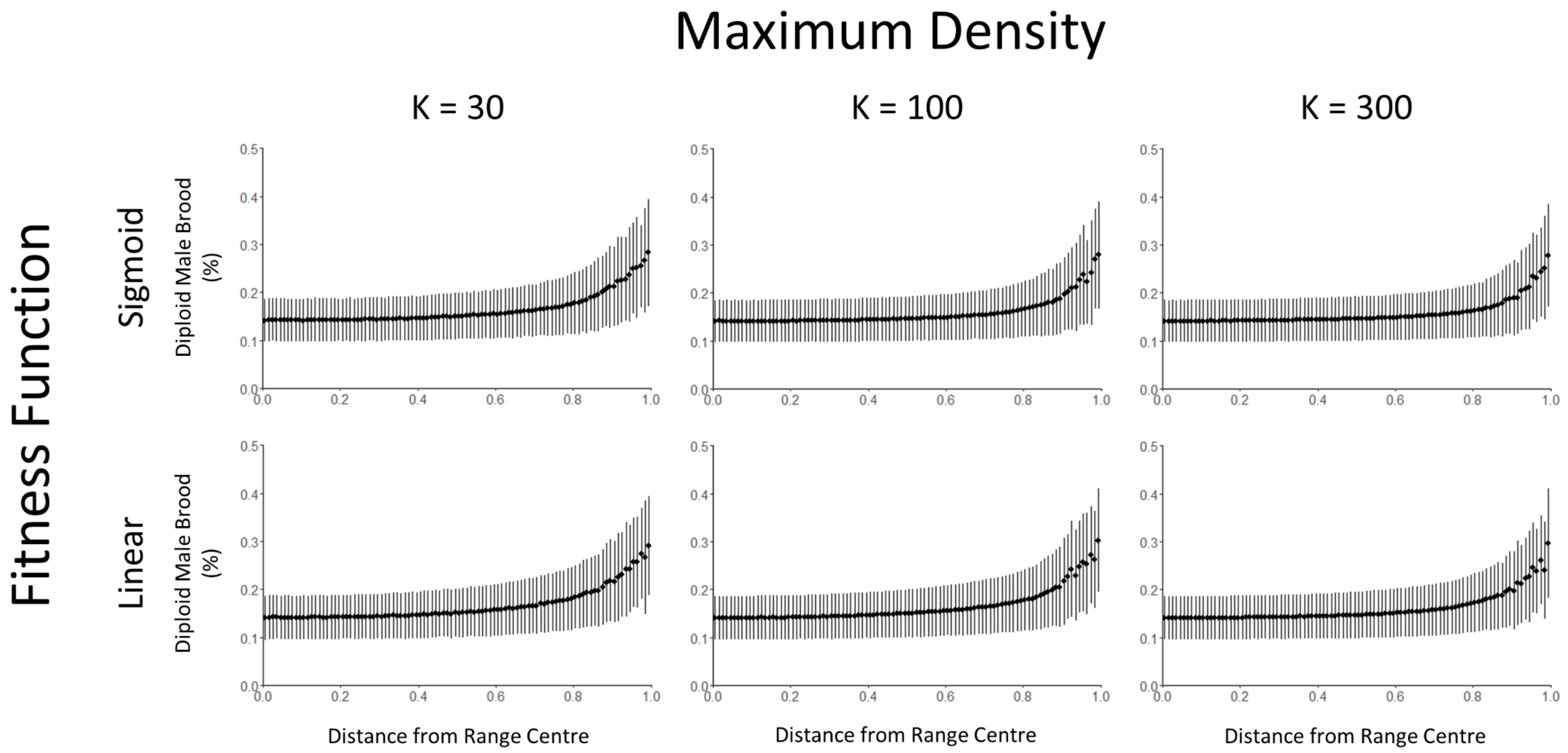

Supplementary Figure 4: Plots of average diploid male brood per colony with distance from range centre for all modelled combinations of the simulation parameter K (maximum colony density: 30, 100 or 300) and two fitness functions (Linear or Sigmoid). In these plots S_A = 3 (average reproductive output) and β = 5 (the dispersal strength). Each point represents the average fitness of colonies for each simulation (n=96) binned at that percentile of distance, and error bars represent standard deviation. Neither K nor the fitness function had significant effects on average rates of diploid male brood at range centres or range edges; that is, the simulation results were not sensitive to variation in these parameters.

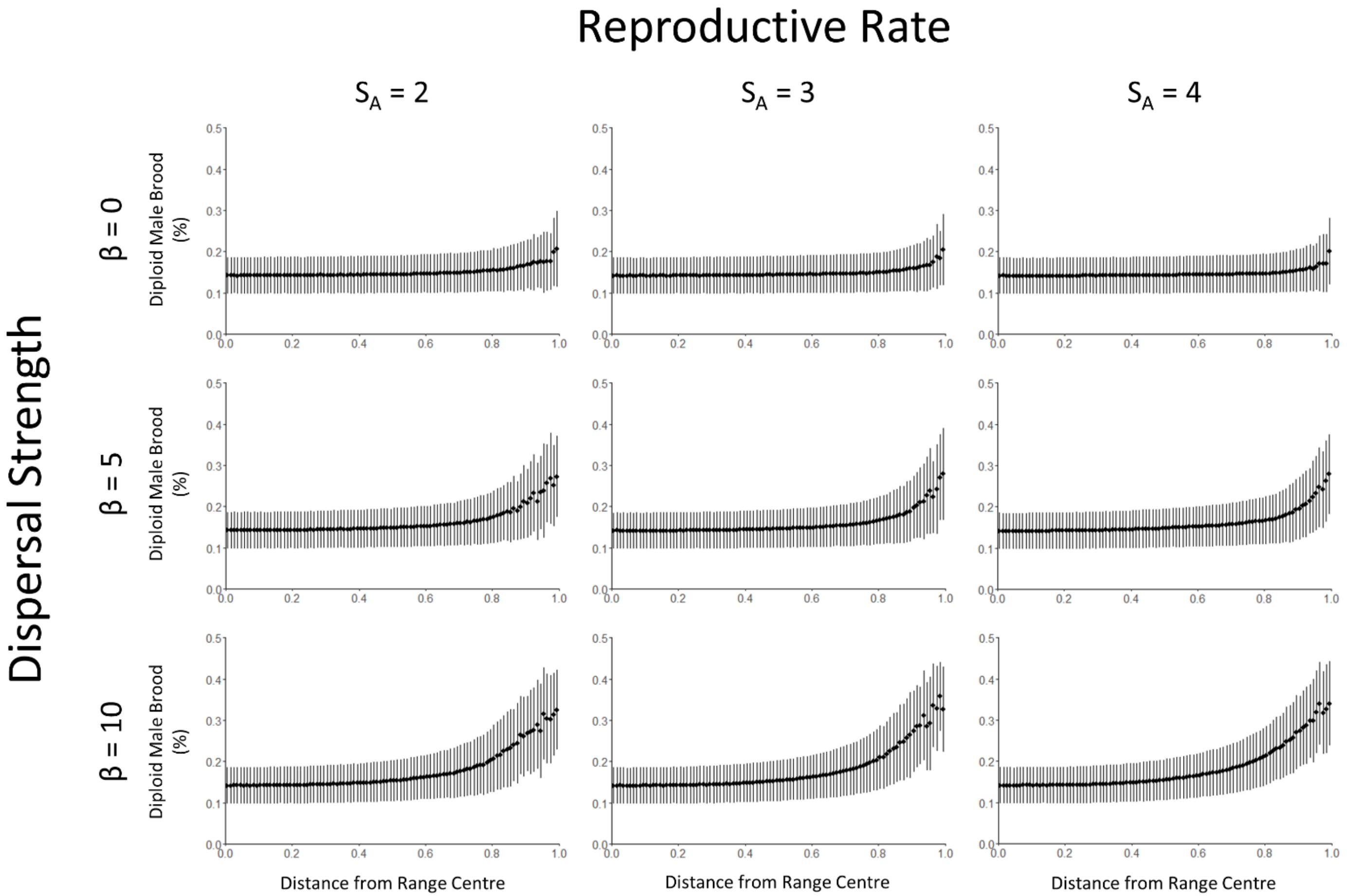

Supplementary Figure 5: Plots of average diploid male brood per colony with distance from range centre for all modelled combinations of the simulation parameters S_A (average reproductive output: 2, 3, 4 swarms) and β (the anisotropic dispersal strength: 0, 5, 10). In these plots K = 100 (maximum colony density) and the fitness function is Sigmoid. Each point represents the average fitness of colonies for each simulation (n=96) binned at that percentile of distance, and error bars represent standard deviation. Rates of diploid male brood in the range centre remain similar across all simulations. For a given value of β, average rates of diploid male brood at range centres or range edges across all values of S_A, indicating that simulation results were not sensitive to average reproductive rate. The magnitude of difference between average diploid male brood at range edges and range centres did vary with dispersal strength (β), however the overall trend of higher brood inviability (i.e. higher diploid male brood proportion) at range edges was consistent across all simulations.

Supplementary Table 1. The nucleotide diversity and polymorphic information content of 2829 genome-wide SNPs in Australia's invasive population of *Apis cerana* for the population as a whole ("All") and per region (Centre, South, Northern Edge and Southern Edge) based on drones sampled at drone congregation areas.

	All	Centre	South	Northern Edge	Southern Edge
Nucleotide Diversity (π)	0.00228	0.00222	0.00223	0.00211	0.00200
N samples (drones)	63	16	16	15	16

Supplementary Table 2. Pairwise measures of population differentiation (Nei's *Fst)* between regions (and 95% CI) for Australia's invasive population of *Apis cerana*, based on 2829 genome-wide SNPs.

Pairwise *Fst*				
	Central	South	Northern Edge	Southern Edge
Central				
South	0.0494			
Northern Edge	0.0650	0.0823		
Southern Edge	0.0952	0.0811	0.1119	
95% Confidence Intervals				
	Central	South	Northern Edge	Southern Edge
Central				
South	0.0434 - 0.0556			
Northern Edge	0.0583 - 0.0718	0.0748 - 0.0903		
Southern Edge	0.0875 - 0.1033	0.0740 - 0.0890	0.1037 - 0.1204	

Supplementary Table 3. Haplotype diversity (*H*) for eight SSR loci in Australia's invasive population of *Apis cerana*, based on males (drones) trapped at mating congregations (N). Values are given for the total population and each region separately. Polymorphic Information Content (PIC), calculated from total population data, and Allele Richness are also given for each locus. Haplotype diversity is presented based on **[A]** total N per region, **[B]** subsampling of each region to match the N of the southern edge region (i.e. N=32) plus 95% CI (105 iterations), and **[C]** subsampling of each region to match the N of the northern edge region (i.e. N=131) plus 95% CI (105 iterations).

	N	**A107**	**Ac1**	**Ac26**	**Ac3**	**B124**	**Ac27**	**Ac32**	**Ac35**	**Average**
PIC	4643	0.32	0.14	0.41	0.36	0.37	0.21	0.50	0.14	0.31
Richness	4643	3	3	4	3	2	2	4	4	3.13
**[A] Haplotype Diversity (*H*)**										
Total Population	4643	0.40	0.15	0.47	0.41	0.50	0.24	0.56	0.14	0.36
Centre	3112	0.37	0.18	0.46	0.41	0.49	0.26	0.59	0.17	0.37
South	722	0.42	0.13	0.52	0.37	0.50	0.13	0.58	0.04	0.34
West	646	0.46	0.08	0.41	0.47	0.49	0.20	0.32	0.12	0.32
Northern Edge	131	0.47	0.10	0.58	0.37	0.50	0.30	0.55	0.20	0.38
Southern Edge	32	0.47	0.12	0.49	0.06	0.47	0.06	0.52	0.06	0.28
**[B] Haplotype Diversity (*H*): down-sampled to southern edge *N***										
Centre	*32*	0.36 (0.15 - 0.49)	0.17 (0 - 0.34)	0.45 (0.25 - 0.59)	0.40 (0.22 - 0.55)	0.48 (0.39 - 0.50)	0.25 (0.06 - 0.41)	0.57 (0.43 - 0.68)	0.16 (0 - 0.33)	
South	*32*	0.41 (0.22 - 0.50)	0.13 (0 - 0.27)	0.50 (0.35 - 0.61)	0.36 (0.17 - 0.53)	0.48 (0.40 - 0.50)	0.13 (0 - 0.27)	0.57 (0.41 - 0.68)	0.04 (0 - 0.17)	
West	*32*	0.44 (0.29 - 0.50)	0.08 (0 - 0.22)	0.39 (0.21 - 0.53)	0.45 (0.17 - 0.58)	0.47 (0.38 - 0.50)	0.19 (0.06 - 0.36)	0.31 (0.12 - 0.48)	0.11 (0 - 0.27)	
Southern Edge	32	0.46	0.12	0.49	0.06	0.47	0.06	0.52	0.06	
**[C] Haplotype Diversity (*H*): down-sampled to northern edge *N***										
Centre	*131*	0.37 (0.28 - 0.44)	0.17 (0.09 - 0.25)	0.46 (0.37 - 0.54)	0.41 (0.32 - 0.49)	0.49 (0.46 - 0.50)	0.26 (0.17 - 0.34)	0.59 (0.52 - 0.64)	0.17 (0.09 - 0.25)	
South	*131*	0.42 (0.35 - 0.47)	0.13 (0.06 - 0.20)	0.51 (0.45 - 0.57)	0.37 (0.28 - 0.45)	0.49 (0.47 - 0.50)	0.13 (0.06 - 0.20)	0.58 (0.51 - 0.64)	0.04 (0 - 0.09)	
West	*131*	0.45 (0.39 - 0.49)	0.08 (0.03 - 0.14)	0.40 (0.32 - 0.47)	0.46 (0.39 - 0.53)	0.48 (0.45 - 0.50)	0.20 (0.12 - 0.28)	0.32 (0.23 - 0.40)	0.12 (0.05 - 0.19)	
Northern Edge	131	0.47	0.10	0.58	0.37	0.50	0.30	0.55	0.20	

Supplementary Table 4. Pairwise measures of population differentiation (Fst) between regions for Australia's invasive population of *Apis cerana*, based on eight SSR loci (N=4643 drones) calculated based on [A] the full dataset, and [B] a dataset that averaged values from random sub-sampling of 32 drones per region, corresponding to the region with the lowest sample size. Confidence intervals (95%) for each set of Fst values are shown.

**[A] Pairwise Fst: full dataset (95% CI)**				
	Centre	South	West	North
Centre				
South	0.0076 (0.0026 - 0.0148)			
West	0.0299 (0.0074 - 0.0502)	0.0269 (0.0099 - 0.0474)		
Northern Edge	0.0166 (0.0023 - 0.0340)	0.0108 (0.0031 - 0.0268)	0.0356 (0.0081 - 0.0755)	
Southern Edge	0.0290 (0.0033 - 0.0486)	0.0094 (-0.0096 - 0.0286)	0.0650 (0.0113 - 0.1091)	0.0050 (-0.0163 - 0.0313)
**[B] Pairwise Fst: subsampling N=32 drones per region (95% CI)**				
Centre				
South	0.0074 (-0.0202 - 0.0521)			
West	0.0296 (-0.0117 - 0.0905)	0.0266 (-0.0133 - 0.0867)		
Northern Edge	0.0165 (-0.0182 - 0.0695)	0.0105 (-0.0197 - 0.0594)	0.0350 (-0.0108 - 0.0993)	
Southern Edge	0.0303 (-0.0035 - 0.0734)	0.0102 (-0.0166 - 0.0471)	0.0652 (0.0195 - 0.1201)	0.0065 (-0.0166 - 0.0381)

Supplementary Table 5. Location of *Apis cerana* drone congregation areas (DCAs) surveyed in Australia's invasive population, whether drones were present at the site (1=yes, 0=no), and the years that the site was surveyed including number of drones sampled per year (empty cells indicate the site was not sampled that year).

Site	Latitude	Longitude	Region	Drones Present	2016	2018	2019
Mulgrave Rd Park, Yungaburra	145.5799	-17.2636	Centre	0			0
Hunter Park, Kuranda	145.6256	-16.8349	Centre	1	103		
Speewah	145.6318	-16.8814	Centre	1			57
Kuranda	145.6481	-16.8092	Centre	1			26
Rainforestation	145.6518	-16.8245	Centre	0			0
Crocodile Park, Kewarra Beach	145.6763	-16.7867	Centre	1	111		
Anne St Park, Smithfield	145.6897	-16.8247	Centre	1	147		
Redlynch Valley position 2	145.6911	-16.9455	Centre	1	35		
Adventure Gully Park, Redlynch	145.6955	-16.9041	Centre	1			94
Redlynch Valley position 1	145.6974	-16.9327	Centre	1	76		
Propellor Court, Trinity Beach	145.6993	-16.8005	Centre	0		0	
Goomboora Park (C)	145.7041	-16.9057	Centre	1	351	180	
Mount Peter	145.7223	-17.0825	Centre	1			4
Sims Esplanade, Yorkeys Knob	145.7265	-16.8106	Centre	1	5		
Edge Hill/Skye Close (C)	145.7372	-16.8994	Centre	1	281	262	158
Ravizza Drive, Edmonton (position 1)	145.7377	-17.0132	Centre	1	209	111	
Mestrez St Park, Mooroobool	145.7389	-16.9401	Centre	1	458	180	
Ravizza Drive, Edmonton (position 2)	145.7406	-17.0129	Centre	1	40		
Lily Creek/Grove St, Cairns Esplanade	145.7623	-16.9149	Centre	1	9		
Davie Park, Cairns Esplanade	145.7629	-16.9096	Centre	1	80		
Patrick Close, Gordonvale	145.7661	-17.0927	Centre	1	135		
Stewart Creek Rd, Daintree	145.3179	-16.2507	Northern Edge	0		0	
Douglas St, Daintree	145.3232	-16.2492	Northern Edge	0		0	
Mossman Gorge	145.3485	-16.4739	Northern Edge	0			0
Daintree River Cruise, Daintree	145.3617	-16.2552	Northern Edge	0		0	
Mossman, showgrounds	145.3653	-16.4625	Northern Edge	1			17
Gwendolyn Rex, Mossman	145.371	-16.4539	Northern Edge	0	0		
Forest Glen Park, Mossman	145.3721	-16.4721	Northern Edge	0		0	
Bamboo	145.392	-16.3518	Northern Edge	0			0
Wonga Beach Park	145.413	-16.3637	Northern Edge	0		0	0
Pinnacle Village Holiday Park, Wonga	145.4197	-16.3295	Northern Edge	1			3
Wharf St Park, Port Douglas	145.4624	-16.4897	Northern Edge	1	13		
Ulysses Ave, Port Douglas	145.467	-16.5249	Northern Edge	1	10	0	
St Crispins, Port Douglas	145.4672	-16.5226	Northern Edge	1		88	
Four Mile Beach Park, Port Douglas	145.4722	-16.5196	Northern Edge	0	0		
Crystal Cascades	145.6815	-16.9615	South	0	0		0
Josephine Falls	145.8591	-17.4381	South	1		6	0
Babinda Boulders	145.8699	-17.3385	South	1	0		7

Paronella Park 1	145.9567	-17.6521	South	0		0	
Paronella Park 2	145.9568	-17.655	South	0		0	
Silkwood Japoon Rd, No 4 Branch	145.9671	-17.7464	South	0		0	
Innisfail Japoon Rd, South Johnstone	145.9841	-17.6124	South	1		2	
Hing St, South Johnstone	145.9924	-17.6029	South	0		0	
South Johnstone River	145.9985	-17.5979	South	0		0	
Wilkie Gray Rd, Boogan	146.0046	-17.6258	South	0		0	
Warrina Botanical Gardens, Innisfail (position 1)	146.0177	-17.5167	South	1	204	180	
Warrina Botanical Gardens, Innisfail (position 2)	146.0235	-17.5189	South	1	35	121	0
Dalrymple Park, Innisfail	146.0374	-17.5236	South	1	120		
Etty Bay Caravan park	146.0893	-17.5575	South	1		1	
Golden Hole	145.853	-17.4479	South	1		45	
Cowley Beach Rd, Cowley Beach	146.1011	-17.7025	South	0		0	
Bilyana Rest Area	145.912	-18.1189	Southern Edge	0			0
Tully Gorge Rd, Tully	145.918	-17.951	Southern Edge	0		0	
Cook St, Tully	145.9212	-17.9282	Southern Edge	0		0	
Venturato Cl, Tully	145.9242	-17.9148	Southern Edge	0		0	
Bike Track, Silky Oak	145.9298	-17.9819	Southern Edge	0		0	
Cricket Field near Country Club, Tully	145.9372	-17.9337	Southern Edge	0		0	
Old Tully Rd, Feluga	145.9691	-17.867	Southern Edge	1		1	
Mitchell Crescent, Merryburn	145.9737	-17.9198	Southern Edge	1		1	
Merryburn Dr, Merryburn	145.9744	-17.9231	Southern Edge	0		0	
Jackson Rd, Shell Pocket	145.9801	-17.8172	Southern Edge	1		2	
Ologhlen Rd, Lower Tully	145.996	-17.9667	Southern Edge	0		0	
Mount Myrtle Rd, East Feluga	146.0016	-17.8773	Southern Edge	0		0	
Millington Rd, El Arish	146.0021	-17.8119	Southern Edge	0		0	
Jaffa Rd, El Arish	146.0035	-17.8005	Southern Edge	0		0	
Oval, El Arish	146.0069	-17.7989	Southern Edge	0		0	
Granadilla Rd, Friday Pocket	146.0316	-17.8434	Southern Edge	0		0	
Tully Heads	146.0558	-18.0066	Southern Edge	0			0
Daveson Rd, Daveson	146.0591	-17.7964	Southern Edge	0		0	
Jackey Jackey St, South Mission Beach	146.0833	-17.9494	Southern Edge	0		0	
Bingil Bay Rd, Bingil Bay	146.0842	-17.8108	Southern Edge	0		0	
Park, South Mission Beach	146.088	-17.9283	Southern Edge	0		0	
Webb Rd, Wongaling Beach	146.093	-17.9049	Southern Edge	0		0	
Ohl Road, Mission Beach	146.0969	-17.858	Southern Edge	0		0	
Boyett Rd, Mission Beach	146.1062	-17.8618	Southern Edge	1		1	
Porter Promenade, Mission Beach	146.1136	-17.8594	Southern Edge	1		27	0
Basalt Gully Park, Mareeba	145.4181	-17.0003	West	1	427	116	7
Mareeba - Mary Andrews Garden Park	145.4186	-16.9799	West	1			38
Mareeba - Bicentennial Lakes	145.4192	-16.9929	West	1			58

Supplementary Table 6. Primers used to amplify a polymorphic fragment of the sex locus (*csd*) of *Apis cerana* and eight single sequence repeats (SSRs). The PCR conditions used for all reactions was as follows: 94°C for 30s, 38 cycles of 94°C for 30s, 56°C for 30s, 72°C for 45s, and then 72°C terminating step for 7 min.

***csd* (sex locus)**					
Forward primer	Sequence (5' to 3')	Reverse primer	Sequence (5' to 3')	Reference	Reference DOI
csd_forA	CAYCRAGAGAACGATCTCG	csd_R2	CCCAAGGTYTAAYCATTATTGG	Gloag et al. 2017, *Nature Ecology and Evolution* 1:011	https://doi.org/10.1038/s41559-016-0011
csd_ForA3/8	GAGRAAGATCTRRAGAAC	csd_R2	CCCAAGGTYTAAYCATTATTGG	Gloag et al. 2017, *Nature Ecology and Evolution* 1:011	
csd_for2	GAAAGAARTTGCAGTARAGATAG	csd_RevA11	CGAGATCGTTCTCTYGATG	Gloag et al. 2017, *Nature Ecology and Evolution* 1:011	
**SSRs (microsatellites)**					
Forward primer	Sequence (5' to 3')	Reverse primer	Sequence (5' to 3')	Reference	Reference DOI
A107-F	CCGTGGGAGGTTTATTGTCG	A107-R	GGTTCGTAACGGATGACACC	Solignac et al. 2003, *Molecular Ecology Notes* 3:307-311	https://doi.org/10.1046/j.1471-8286.2003.00436.x
B124-F	GCAACAGGTCGGGTTAGAG	B124-R	CAGGATAGGGTAGGTAAGCAG	Solignac et al. 2003, *Molecular Ecology Notes* 3:307-311	
Ac1-F	GCTACACTACCGCGACCTGCA	Ac1-R	TACGCTCCGTTAGTCCCGCTG	Takahashi et al. 2009, *Molecular Ecology Resources* 9:819-821	https://doi.org/10.1111/j.1755-0998.2009.02268.x
Ac26-F	AACCTTCTTCGCCACCTCCAA	Ac26-R	GTCTGAACGAAAGAAAGAGCA	Takahashi et al. 2009, *Molecular Ecology Resources* 9:819-821	
Ac3-F	TTGTCTTGCTCATTGCTTCCA	Ac3-R	AGGAGACGGTTCCACGGAAAG	Takahashi et al. 2009, *Molecular Ecology Resources* 9:819-821	
Ac27-F	CTTTCTCTCTGCCTTTCTTTC	Ac27-R	ACGCATGGAATGGAGAAACTG-	Takahashi et al. 2009, *Molecular Ecology Resources* 9:819-821	-
Ac32-F	GTCGCCATAGTCCTCTACCAA	Ac32-R	CGACGATATGAGAATAGGAGT	Takahashi et al. 2009, *Molecular Ecology Resources* 9:819-821	
Ac35-F	TTCCAGCAGGAAGTGACGGTG	Ac35-R	CGGGAAACTCGTCATTTTCG	Takahashi et al. 2009, *Molecular Ecology Resources* 9:819-821	

Supplementary Table 7. Allele richness and frequencies for eight SSR loci and the sex locus (*csd*) in Australia's invasive population of *Apis cerana*, based on males (drones) trapped at mating congregations (*N*). Cells that are not filled indicate 0 observed individuals with that allele, such that number of filled cells per locus indicate allele richness per region. Individual genotypes are given in Supplementary Data 1.

Locus	Alleles	Centre	West	South	Northern Edge	Southern Edge
	N	**3112**	**646**	**722**	**131**	**32**
A107	169	0.2439	0.3502	0.3023	0.3846	0.3846
	172	0.7557	0.6498	0.6977	0.6154	0.6154
	175	0.0004				
Ac1	200	0.0003				
	202	0.9030	0.9568	0.9303	0.9492	0.9355
	204	0.0967	0.0432	0.0697	0.0508	0.0645
Ac26	132	0.0539	0.0153	0.0647	0.0263	
	138	0.2201	0.2428	0.3010	0.3772	0.4194
	144	0.6981	0.7317	0.6235	0.5263	0.5806
	148	0.0278	0.0102	0.0118	0.0702	
Ac3	314	0.7347	0.6858	0.7713	0.7757	0.9677
	316	0.0551	0.0702	0.0660	0.1121	
	318	0.2102	0.2440	0.1628	0.1121	0.0323
B124	217	0.5567	0.4159	0.5431	0.5133	0.6129
	219	0.4433	0.5841	0.4569	0.4867	0.3871
Ac27	122	0.1559	0.1135	0.0704	0.1860	0.0323
	136	0.8441	0.8865	0.9296	0.8140	0.9677
Ac32	72	0.5561	0.8117	0.5768	0.5625	0.5484
	74	0.2893	0.1377	0.2584	0.3594	0.4194
	76	0.1132	0.0443	0.1117	0.0469	
	80	0.0414	0.0063	0.0531	0.0313	0.0323
Ac35	123	0.0013				
	125	0.9088	0.9376	0.9790	0.8915	0.9677
	127	0.0320	0.0080	0.0056	0.0465	0.0323
	131	0.0579	0.0544	0.0154	0.0620	
csd	1	0.1539	0.1786	0.1771	0.1163	0.4375
	2	0.2050	0.1276	0.1980	0.1318	0.5000
	3	0.1247	0.1244	0.1130	0.0775	
	4	0.1612	0.2073	0.1325	0.0310	0.0313
	5	0.1751	0.1515	0.1660	0.2636	
	6	0.0899	0.1196	0.0948	0.1395	
	7	0.0902	0.0909	0.1185	0.2403	0.0313

Supplementary Table 8. The sample year, location (lat, long), number of workers genotyped at *csd* and inferred % diploid brood per colony that were males (i.e. diploid male production, DMP) for 75 colonies of *Apis cerana* collected from Australia's invasive population. The full genotype data for each worker used to calculate DMP is given in Supplementary Data 4.

Colony ID	Year	Latitude	Longitude	*N* workers genotyped	DMP
IP785	2012	-16.461445	145.372665	48	0.4074
IP658	2012	-16.490554	145.464635	23	0.2333
IP567	2012	-16.91934	145.75959	31	0.1389
IP635	2012	-16.89091	145.6943	31	0.2619
IP639	2012	-16.91677	145.75893	31	0.0000
IP645	2012	-17.00323	145.44597	31	0.2250
IP662	2012	-16.97098	145.74814	31	0.4259
IP694	2012	-17.27237	145.48212	31	0.2791
IP723	2012	-16.8954	145.6956	31	0.1842
IP726	2012	-16.99011	145.42614	31	0.3261
IP741	2012	-17.0096	145.73955	31	0.0606
IP742	2012	-16.81554	145.71943	31	0.1622
IP745	2012	-17.04438	145.73845	31	0.0606
IP746	2012	-16.85893	145.53589	31	0.2791
IP616	2012	-16.783593	145.697559	29	0.1714
Mareeba-2012	2012	-16.999028	145.422683	31	0.0313
Tolga-2012	2012	-17.223334	145.478673	29	0.2162
Edmond-2012	2012	-17.021912	145.730694	30	0.2683
15C.15	2012	-16.90819	145.68857	96	0.4607
15C.36	2015	-16.79716	145.69143	73	0.0759
15C.22	2015	-16.79661	145.69158	95	0.1667
15C.23	2015	-16.79374	145.67234	127	0.1361
15C.21	2015	-16.78721	145.69312	64	0.1467
15C.6	2015	-16.906086	145.75674	94	0.0505
15C.12	2015	-16.803324	145.719252	95	0.2149
15C.17	2015	-16.79718	145.6747	191	0.0591
15C.18	2015	-16.8872	145.7478	180	0.1220
15C.51	2015	-16.93773	145.76958	39	0.0488
15C.8	2015	-16.782517	145.680213	37	0.0513
15C.32	2015	-16.84528	145.7384	96	0.2441
15C.34	2015	-17.19835	145.52398	96	0.1028
15C.52	2015	-16.91381	145.74837	96	0.1351

15C.53	2015	-16.905302	145.745619	95	0.2339
15C.5	2015	-16.79341	145.67674	96	0.1028
15C.13	2015	-16.931442	145.772738	96	0.1933
15C.48	2015	-16.87107	145.67962	96	0.1028
15C.39	2015	-16.87458	145.44167	94	0.4088
Wonga-2015	2015	-16.357103	145.41466	96	0.4828
15C.39	2015	-16.87458	145.44167	31	0.4038
15C.34	2015	-17.19835	145.52398	31	0.1389
15C.19	2015	-17.1099	145.7735	17	0.2609
15C.10	2015	-16.60038	145.51649	20	0.1304
15C.9	2015	-17.09163	145.79275	23	0.2069
19C.16	2019	-16.94601	145.77272	41	0.1087
19C.6	2019	-16.32042	145.41997	35	0.0000
19C.12	2019	-16.5619	145.39039	38	0.1163
19C.13	2019	-16.46016	145.37622	37	0.0976
19C.14	2019	-16.46016	145.37622	36	0.0769
19C.22	2019	-16.66986	145.32511	39	0.0930
19C.23	2019	-16.49169	145.39123	39	0.2353
19C.4	2019	-16.99043	145.41668	32	0.2381
19C.5	2019	-17.26804	145.49006	37	0.0750
19C.8	2019	-17.27132	145.58567	39	0.1136
19C.9	2019	-16.91802	145.41942	33	0.2500
19C.17	2019	-17.528787	146.006042	39	0.0488
19C.18	2019	-17.184053	145.886532	37	0.0513
19C.26	2019	-17.20376	145.55854	38	0.1915
19C.24	2019	-16.8965	145.73972	37	0.0513
19C.7	2019	-16.90198	145.71065	28	0.1250
19C.10	2019	-16.80404	145.72074	28	0.3488
19C.1	2019	-16.90413	145.69349	31	0.2250
19C.2	2019	-17.01696	145.74369	31	0.2250
19C.3	2019	-16.83828	145.69407	31	0.0882
19C.11	2019	-16.93875	145.72613	31	0.1842
19C.15	2019	-16.90022	145.73202	31	0.1842
19C.19	2019	-17.09007	145.7869	31	0.0882
19C.20	2019	-16.78142	145.67822	27	0.1818
19C.21	2019	-16.99316	145.7365	31	0.1389

19C.27	2019	-16.78702	145.68787	31	0.2051
19C.28	2019	-16.8489	145.73732	27	0.0357
19C.29	2019	-16.74789	145.66742	31	0.1389
19C.30	2019	-16.79737	145.67378	31	0.1143
19C.31	2019	-17.03154	145.74085	31	0.1389
Silky Oak-2020	2020	-17.980804	145.946302	76	0.3920
Cardwell-2022	2022	-18.27475	146.0086111	31	0.2955

Supplementary References

1. Oldroyd BP, Lawler SH, Crozier RH. Do feral honey bees (*Apis mellifera*) and regent parrots (*Polytelis anthopeplus*) compete for nest sites? *Australian Journal of Ecology* **19**, 444-450 (1994).

2. Ding G, Xu H, Oldroyd BP, Gloag RS. Extreme polyandry aids the establishment of invasive populations of a social insect. *Heredity* **119**, 381-387 (2017).

3. Gloag RS, Christie JR, Ding G, Stephens RE, Buchmann G, Oldroyd BP. Workers' sons rescue genetic diversity at the sex locus in an invasive honey bee population. *Molecular Ecology* **28**, 1585-1592 (2019).

4. Utaipanon P, Holmes MJ, Chapman NC, Oldroyd BP. Estimating the density of honey bee (*Apis mellifera*) colonies using trapped drones: Area sampled and drone mating flight distance. *Apidologie* **50**, 578-592 (2019).

5. McNally L, Schneider S. Drone production and drone comb utilization in colonies of the African honey bee, *Apis mellifera scutellata* Lepeletier, in Africa. *Apidologie* **25**, 547-556 (1994).

6. Villa JD. Swarming Behavior of Honey Bees (Hymenoptera: Apidae) in Southeastern Louisiana. *Annals of the Entomological Society of America* **97**, 111-116 (2004).

7. Bozina K. How long does the queen live? *Pchelovodstvo* **38**, 13 (1961).

8. Seeley TD. Life history strategy of the honey bee, *Apis mellifera*. *Oecologia* **32**, 109-118 (1978).
