# Supplementary material for: Serial founder effects slow range expansion in an invasive social insect
Source: Nat Commun. 2024 Apr 29;15:3608. doi: 10.1038/s41467-024-47894-1 (PMC11058855; doi:10.1038/s41467-024-47894-1)
Supplement: Supplementary file 3 — Reporting Summary [file 41467_2024_47894_MOESM3_ESM.pdf]

nature portfolio

Corresponding author(s): Rosalyn Gloag

Last updated by author(s): 25/03/24

Reporting Summary

Nature Portfolio wishes to improve the reproducibility of the work that we publish. This form provides structure for consistency and transparency in reporting. For further information on Nature Portfolio policies, see our Editorial Policies and the Editorial Policy Checklist.

Statistics

For all statistical analyses, confirm that the following items are present in the figure legend, table legend, main text, or Methods section.

n/a	Confirmed	
☐	☒	The exact sample size (*n*) for each experimental group/condition, given as a discrete number and unit of measurement
☐	☒	A statement on whether measurements were taken from distinct samples or whether the same sample was measured repeatedly
☐	☒	The statistical test(s) used AND whether they are one- or two-sided<br>*Only common tests should be described solely by name; describe more complex techniques in the Methods section.*
☒	☐	A description of all covariates tested
☒	☐	A description of any assumptions or corrections, such as tests of normality and adjustment for multiple comparisons
☐	☒	A full description of the statistical parameters including central tendency (e.g. means) or other basic estimates (e.g. regression coefficient) AND variation (e.g. standard deviation) or associated estimates of uncertainty (e.g. confidence intervals)
☐	☒	For null hypothesis testing, the test statistic (e.g. *F*, *t*, *r*) with confidence intervals, effect sizes, degrees of freedom and *P* value noted<br>*Give P values as exact values whenever suitable.*
☒	☐	For Bayesian analysis, information on the choice of priors and Markov chain Monte Carlo settings
☒	☐	For hierarchical and complex designs, identification of the appropriate level for tests and full reporting of outcomes
☒	☐	Estimates of effect sizes (e.g. Cohen's *d*, Pearson's *r*), indicating how they were calculated

Our web collection on statistics for biologists contains articles on many of the points above.

Software and code

Policy information about availability of computer code

Data collection	Our simulation model was written and run in R version 4.0.4, and the code can be found on github (https://github.com/Thomas-Hagan/BeeSimulation.git). DOI: 10.5281/zenodo.10783818.
Data analysis	All statistical tests were performed using R (core team, 4.0.4). Packages used: dartR v2, ade4 v1.7-22, hierfstat v0.5-11, ggplot2 v3.4.1.

For manuscripts utilizing custom algorithms or software that are central to the research but not yet described in published literature, software must be made available to editors and reviewers. We strongly encourage code deposition in a community repository (e.g. GitHub). See the Nature Portfolio guidelines for submitting code & software for further information.

Data

Policy information about availability of data

All manuscripts must include a data availability statement. This statement should provide the following information, where applicable:

- Accession codes, unique identifiers, or web links for publicly available datasets
- A description of any restrictions on data availability
- For clinical datasets or third party data, please ensure that the statement adheres to our policy

Data Availability Statement: The DArTseq raw data files generated in this study have been deposited in the NCBI Sequence Read Archive, BioProject PRJNA1090620. The reference genome used in this study (ACSNU-2.0) is available in GenBank under accession code GCA_001442555.1. The simulation data generated in this study is available at Figshare.com (10.6084/m9.figshare.25395418). All other data supporting the findings of this study are available within the paper and its

Supplementary Information and Data (see also "Software and Code" above). Underlying data for Figure 1A are provided in Supplementary Table 5. Underlying data for Figure 1B are provided in Supplementary Data 1. Underlying data for Figure 2A-D are provided in Supplementary Table 8. Underlying Data for Supplementary Figure 1A are provided in Supplementary Data 2 and 3, and data used to produce Supplementary Figure 1B are provided in Supplementary Data 1. Summary data for Figure 3C is included in Supplementary Data 5.

Research involving human participants, their data, or biological material

Policy information about studies with human participants or human data. See also policy information about sex, gender (identity/presentation), and sexual orientation and race, ethnicity and racism.

Reporting on sex and gender	NA
Reporting on race, ethnicity, or other socially relevant groupings	NA
Population characteristics	NA
Recruitment	NA
Ethics oversight	NA

Note that full information on the approval of the study protocol must also be provided in the manuscript.

Field-specific reporting

Please select the one below that is the best fit for your research. If you are not sure, read the appropriate sections before making your selection.

☐ Life sciences ☐ Behavioural & social sciences ☒ Ecological, evolutionary & environmental sciences

For a reference copy of the document with all sections, see nature.com/documents/nr-reporting-summary-flat.pdf

Ecological, evolutionary & environmental sciences study design

All studies must disclose on these points even when the disclosure is negative.

Study description	The study uses three datasets.<br>(1) We sampled A. cerana drones (haploid males) at natural drone congregation areas (DCAs) to assess population level genetic diversity of the sex locus (csd). For each subpopulation we bootstrapped a range of expected Haplotype diversities, to which we compared our observed Haplotype diversity and determined whether this lay within the 95% most common values (Fig 1). We then also performed nucleotide diversity analyses (Supp Table 1), PCAs (Supp Fig 1) and calculated pairwise Fst's (Supp Tables 2-3) for microsatellite and SNP (DArT-Seq: reduced representation) data, to see if subpopulations differed in neutral genetic diversity.<br>(2) We sampled colonies and inferred the proportion of diploid males, DMP (inviable diploid brood) each colony produced based on the sex allele distribution present in the viable diploid brood (i.e. workers). We then determined the relationship between DMP of colonies and their distance from the range centre using a regression (where distance was normalised across years to account for population spread; Fig 2).<br>(3) We performed agent-based simulations of an invading and expanding honey bee population to determine whether the increase in diploid male production (and decrease in fitness) at range edges observed in our empirical data from (1) and (2) is expected to slow the rate of population spread. We determined spatial patterns in diploid male production across a range of parameter values (Fig S2 and S3). We then repeated the simulations for a population where the simulated locus was a neutral locus (instead of a homozygous-lethal locus) and compared the distance these simulated populations reached after 20 generations (Fig 3).
Research sample	(1) Drones (males) trapped at mating congregations were chosen to examine population level genetic diversity of Apis cerana as drones from many nearby colonies all congregate in specific locations to mate with queens, allowing the sampling of representative local population genetic diversity. We found and sampled these DCAs across the invasive range of A. cerana in Australia (Fig 1). These samples may have varied in age, but drones do not live for longer than 8 weeks, so they provide a recent snapshot of population diversity.<br>(2) Colonies of Apis cerana were chosen to examine the fitness effects of low diversity at the sex locus i.e. the proportion of viable female brood produced by a queen. Workers and queens (females) were used to calculate the average diploid male production in a colony (where diploid males are inviable in honey bees). A minimum of 17 workers and maximum of 191 workers were used to estimate this proportion of diploid male brood for each colony, depending on the number of workers available from field collections. Colonies were collected from throughout the invasive range of A. cerana (Fig 2).
Sampling strategy	(1) In the case of drone collections, repeated sampling of DCAs during a field trip was performed until sample sizes exceeded 100 drones in a single collection period. Bootstrapping analyses indicate that local populations were represented by ~100 drones.. Where this was not the case (i.e., samples were collected <100 drones despite repeated efforts), the DCAs were at range edge populations where low sample numbers are expected and representative. Subpopulations included as many samples as possible, usually well over the prerequsite 100 drones. To account for sample size differences, we performed bootstrap analyses by sample on csd analysis and microsatellite pairwise Fst. SNPs were then obtained for a subsample of drones via DArTSeq, Diversity Array Technologies, Canberra.

	SNP analysis was performed using a PCA on a random sample of 15-16 drones per region (n=63 total).<br>(2) In the case of colony collections, all possible colonies collected in the target years (2012, 2015, 2019-2022) were used.
Data collection	Data collection for field samples were undertaken by Thomas Hagan, Guiling Ding, Ros Gloag and Ben Oldroyd, along with some volunteers per field trip (in acknowledgements). In the field collections of drones at DCAs and colonies from various locations were recorded with the date located, position found, and general condition of samples. Analysis of genetics were performed by Ros Gloag, Thomas Hagan, Guiling Ding and Gabrielle Buchmann, who each called alleles of microsatellites and sex locus fragments in Genemapper after PCR. DArTSeq was performed on a subset of drone samples at Diversity Array Technologies, Canberra, with DNA extractions for these samples performed by Thomas Hagan.
Timing and spatial scale	Field trips by the authors occurred in August-September 2015 (4 weeks), July-August 2016 (5 weeks), April-May 2018 (4 weeks), July-August 2019 (6 weeks), October-November 2019 (3 weeks) and April 2021 (3 weeks). Other samples were collected by Queensland Department of Agriculture Fisheries & Forestries throughout the years 2012-2022. This study takes place over a range of 150 km from the centre of the Invasive A. cerana population, Cairns and 10 years of sampling of the expanding population.
Data exclusions	No data was excluded from our study.
Reproducibility	Our study reports on and analyses genetic diversity in a natural population. We were able to observe consistent trends at different range edges of our study population, though given the logistic challenges of this field study we focused on the southern range edge when sampling DCAs.
Randomization	Randomization is not relevant to our study. Our study investigated changes in genetic diversity across space in a natural population of honey bees.
Blinding	Blinding is not relevant to our study. Our study investigated changes in genetic diversity across space in a natural population of honey bees.

Did the study involve field work? ☒ Yes ☐ No

Field work, collection and transport

Field conditions	All fieldwork was conducted in tropical Far North Queensland, Australia, within 150km of Cairns (16.9203° S, 145.7710° E).
Location	Field trips by the authors to the Cairns, Queensland region (16.9203° S, 145.7710° E) occurred in August-September 2015 (4 weeks), July-August 2016 (5 weeks), April-May 2018 (4 weeks), July-August 2019 (6 weeks), October-November 2019 (3 weeks) and April 2021 (3 weeks). Other samples were collected by Queensland Department of Agriculture Fisheries & Forestries throughout the years 2012-2022. Maps showing the collection location of all samples are provided in the main text (drones: Fig 1, colonies: Fig 2) and precise coordinates of sample locations are given in Supplementary Tables 5 & 8.
Access & import/export	All sampling was conducted with permission from Queensland Department of Agriculture Fisheries and Forestries. Some sampling occurred in National Parks under permit WITK18775018. Our sampling methods for trapping Apis cerana drones are species-specific because they target the species' sex pheromone and congregation times; there is zero by-catch of native or other species.
Disturbance	Our study caused no significant disturbance. Apis cerana is an invasive species. Our sampling methods targeted Apis cerana only. All access to sampling sites was via roads.

Reporting for specific materials, systems and methods

We require information from authors about some types of materials, experimental systems and methods used in many studies. Here, indicate whether each material, system or method listed is relevant to your study. If you are not sure if a list item applies to your research, read the appropriate section before selecting a response.

Materials & experimental systems

n/a	Involved in the study	
☒	☐	Antibodies
☒	☐	Eukaryotic cell lines
☒	☐	Palaeontology and archaeology
☐	☒	Animals and other organisms
☒	☐	Clinical data
☒	☐	Dual use research of concern
☒	☐	Plants

Methods

n/a	Involved in the study	
☒	☐	ChIP-seq
☒	☐	Flow cytometry
☒	☐	MRI-based neuroimaging

Animals and other research organisms

Policy information about studies involving animals; ARRIVE guidelines recommended for reporting animal research, and Sex and Gender in Research

Laboratory animals	NA
Wild animals	The Asian honey bee (also called Asian hive bee), Apis cerana. Both drones (males) from drone congregation areas and workers (females) sampled from colonies. All collected individuals were killed either by placing directly onto 100% ethanol or placing into the freezer (-20C). Such methods align with the AVMA guidelines for terrestrial invertebrates.
Reporting on sex	Our samples include both drones (males) from drone congregation areas and workers (females) sampled from colonies. Male and female datasets are analyzed separately; see main text.
Field-collected samples	We collected Asian honey bee Apis cerana workers (females) and drones (males) during fieldwork in the Cairns region of Australia (16.9203° S, 145.7710° E). See "Wild animals".
Ethics oversight	No ethical approval or guidance was required. No such approval is required for work with insects in Australia.

Note that full information on the approval of the study protocol must also be provided in the manuscript.
