# Supplementary material for: Serial founder effects slow range expansion in an invasive social insect
Source: Nat Commun. 2024 Apr 29;15:3608. doi: 10.1038/s41467-024-47894-1 (PMC11058855; doi:10.1038/s41467-024-47894-1)
Supplement: Supplementary file 4 — Description of Additional Supplementary Files [file 41467_2024_47894_MOESM4_ESM.pdf]

Description of Additional Supplementary Files

File Name: Supplementary Data 1

Description: The genotypes of 4642 *Apis cerana* drones sampled at drone congregation areas (DCAs) within Australia's invasive population, including the sample year, site and population region. Loci with missing data are indicated with zeros. Details for loci are given in Table S1. The *csd alleles* in this population are numbered 1-7.

File Name: Supplementary Data 2

Description: RAW DArTseq data for *Apis cerana* drones used in this publication. File Name: Supplementary Data 3. Description: Sample ID and population location for males (drones) sequenced for SNPs at Diversity Arrays Technology (DArT), Canberra Australia, corresponding to raw data in Supplementary Data 4.

File Name: Supplementary Data 4

Description: The csd (sex locus) genotype data of workers sampled from 75 colonies of *Apis cerana* in Australia's invasive population, where only seven *csd alelles* (1-7) are present in the population. Details of Colony IDs are given in Supplementary Data 7.

File Name: Supplementary Data 5

Description: The maximum distances reached by simulated colonies in simulations (Sigmoid fitness function, K = 100, S_A = 3) for β = 0, 5, 10 and Homozygous Lethal or Neutral Loci. Data used in Figure 3C.
